# Supplementary material for: Cell-specific measurements show nitrogen fixation by particle-attached putative non-cyanobacterial diazotrophs in the North Pacific Subtropical Gyre
Source: Nat Commun. 2022 Nov 15;13:6979. doi: 10.1038/s41467-022-34585-y (PMC9666432; doi:10.1038/s41467-022-34585-y)
Supplement: Supplementary file 1 — Supplementary Information [file 41467_2022_34585_MOESM1_ESM.pdf]

Supplementary Information for Cell-specific measurements show nitrogen fixation by particle-attached putative non-cyanobacterial diazotrophs in the North Pacific Subtropical Gyre

*Supplementary Figures*

| **Station - treatment** | **Average NFR (nmol N $l^{-1}$ $d^{-1}$)** | **Stdev NFR (nmol N $l^{-1}$ $d^{-1}$)** | **LOD (nmol N $l^{-1}$ $d^{-1}$)** | **MQR (nmol N $l^{-1}$ $d^{-1}$)** | **Detected?** | **Quantified?** |
|---|---|---|---|---|---|---|
| S5 - light | 0.51 | 0.11 | 0.19 | 0.13 | yes | yes |
| S5 - dark | 0.35 | 0.14 | 0.16 | 0.15 | yes | yes |
| S10 - light | 0.19 | 0.13 | 0.12 | 0.13 | yes | yes |
| S10 - dark | 0.13 (x) | 0.07 | 0.32 | 0.10 | No | yes |
| S14 - light | 0.23 (x) | 0.12 | 0.29 | 0.13 | No | yes |
| S14 - dark | 0.06 (x) | 0.04 | 0.08 | 0.05 | No | yes |
| S17 - light | 0.10 (x) | 0.04 | 0.33 | 0.09 | No | yes |
| S17 -dark | 0.05 | 0.03 | 0.29 | 0.08 | No | No |
| S20 - light | 0.15 (x) | 0.12 | 0.24 | 0.10 | No | yes |
| S20 - dark | 0.00 | 0.02 | 0.15 | 0.05 | No | No |
| S22 - light | 0.12 (x) | 0.02 | 0.17 | 0.07 | No | yes |
| S22 - dark | 0.06 (x) | 0.02 | 0.18 | 0.03 | No | yes |
| S23 - light | 0.38 | 0.07 | 0.16 | 0.08 | yes | yes |

*Supplementary Table 1*: Summary of natural-light and all-dark $N_2$ fixation rates (NFR). Average NFR is the average rate (nmol N $l^{-1}$ $d^{-1}$) from biological replicate (n=3) incubations and the associated standard deviation. Limit of detection (LOD) and minimum quantifiable rate (MQR) were calculated for each location and light treatment. Average NFR that were below the LOD (a conservative estimate) but above the MQR are denoted with x.

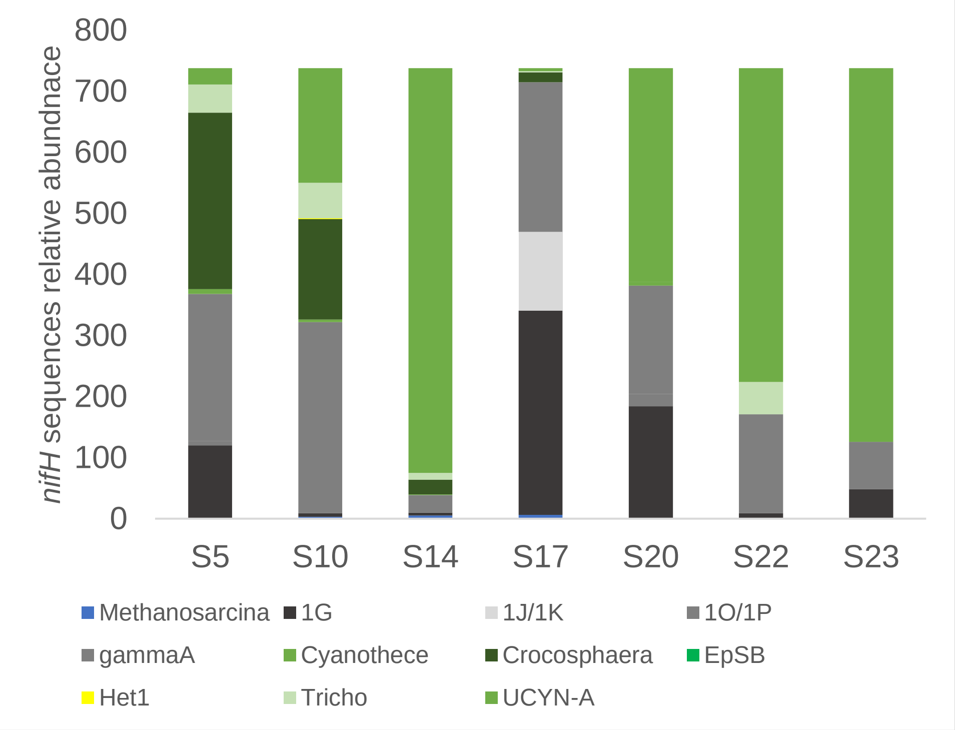


*Supplementary Figure 1: Relative abundance of nifH* gene sequences rarified to minimum sequence recovery (738 sequences). The various shades of green correspond to cyanobacterial *nifH* sequences while grey shades correspond to bacterial non-cyanobacterial diazotrophs and blue (S17) is an archaeal non-cyanobacterial diazotroph. EpSB – *c* (Schvarcz et al., 2022). Source data are provided as a Source Data file.

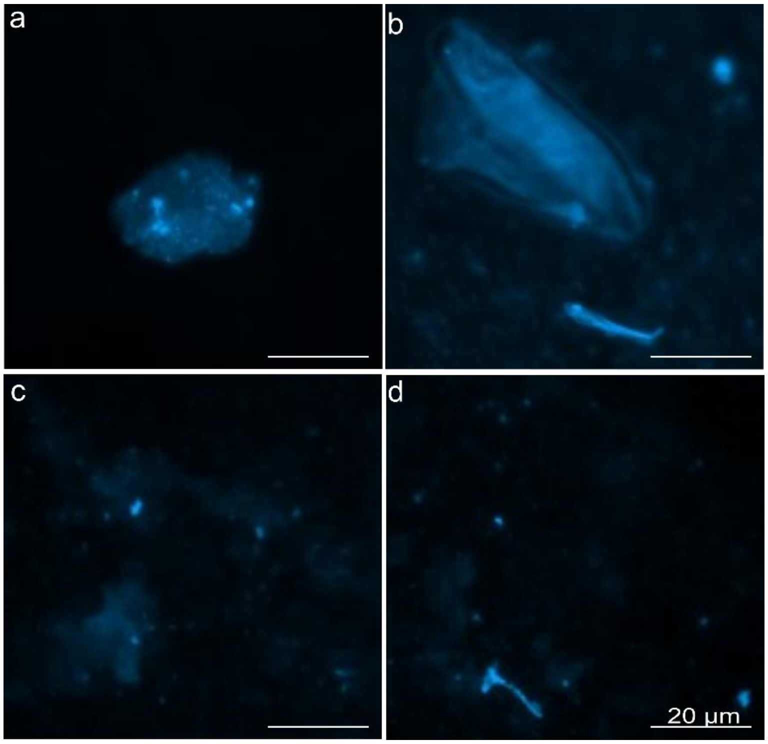


*Supplementary Figure 2:* Examples of types of particles with attached cells. Images are DAPI stained samples corresponding to the samples used for nanoSIMS analysis. Scale bar is 20 µm. Cells are visible in bright blue while the particle matrix can be seen as hazy blue. Particles consisted of densely packed particles (a and b) and loose aggregates (c and d). Particles were counted and observed along 3 transects of filter pieces from each station ranging from 86 to 392 particles per station revealing a variety of morphologies represented by the example images here.

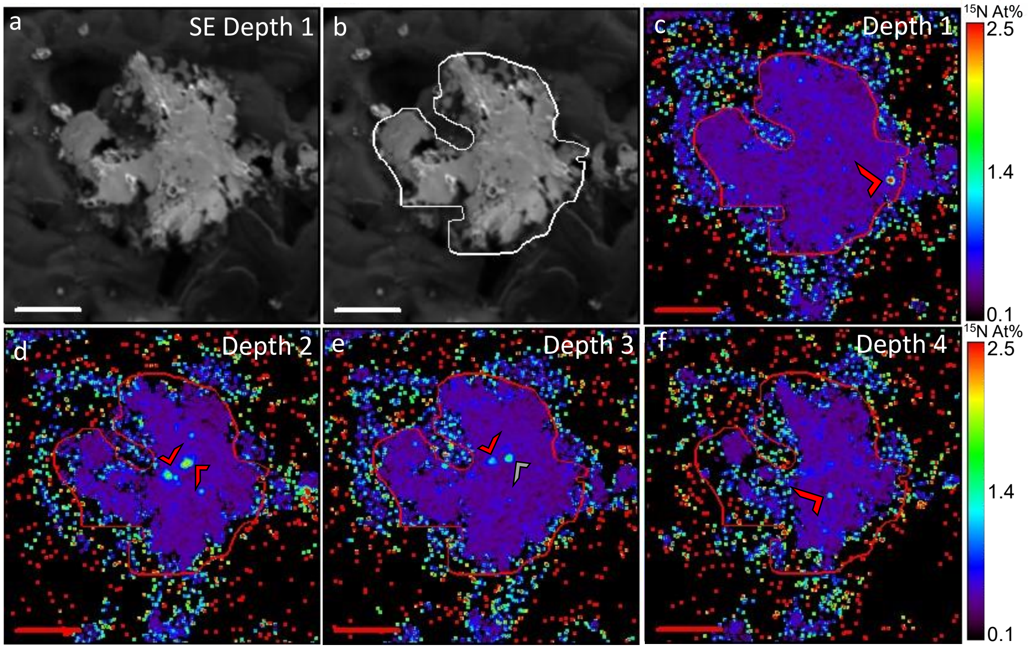


*Supplementary Figure 3:* Particle depth profile showing $^{15}N$ At%. Secondary electron image of particle (a) and outline of particle (b) corresponding to outlines show in (c-f). A particle analyzed at multiple depths showing several additional putative NCD cells (red arrows) that were not identifiable from the particle surface (c-f). The grey arrow (depth 3, image e) is likely the same NCD ROI that was identified in depth 2 (d). A total of 4 particle depth profiles were analyzed of which 3 were found with additional NCD ROIs within the particle. Depth profiles demonstrate the likelihood of missing putative NCD cells on the interior or underside of particles. The scale bar is 5 µm.
