# Supplementary material for: Cell-specific measurements show nitrogen fixation by particle-attached putative non-cyanobacterial diazotrophs in the North Pacific Subtropical Gyre
Source: Nat Commun. 2022 Nov 15;13:6979. doi: 10.1038/s41467-022-34585-y (PMC9666432; doi:10.1038/s41467-022-34585-y)
Supplement: Supplementary file 3 — Reporting Summary [file 41467_2022_34585_MOESM3_ESM.pdf]

nature research

Corresponding author(s): Jonathan Zehr, Xavier Mayali

Last updated by author(s): 2022-26-10

Reporting Summary

Nature Research wishes to improve the reproducibility of the work that we publish. This form provides structure for consistency and transparency in reporting. For further information on Nature Research policies, see our Editorial Policies and the Editorial Policy Checklist.

Statistics

For all statistical analyses, confirm that the following items are present in the figure legend, table legend, main text, or Methods section.

n/a	Confirmed	
☐	☒	The exact sample size (*n*) for each experimental group/condition, given as a discrete number and unit of measurement
☐	☒	A statement on whether measurements were taken from distinct samples or whether the same sample was measured repeatedly
☒	☐	The statistical test(s) used AND whether they are one- or two-sided<br>*Only common tests should be described solely by name; describe more complex techniques in the Methods section.*
☒	☐	A description of all covariates tested
☐	☒	A description of any assumptions or corrections, such as tests of normality and adjustment for multiple comparisons
☐	☒	A full description of the statistical parameters including central tendency (e.g. means) or other basic estimates (e.g. regression coefficient) AND variation (e.g. standard deviation) or associated estimates of uncertainty (e.g. confidence intervals)
☒	☐	For null hypothesis testing, the test statistic (e.g. *F*, *t*, *r*) with confidence intervals, effect sizes, degrees of freedom and *P* value noted<br>*Give P values as exact values whenever suitable.*
☒	☐	For Bayesian analysis, information on the choice of priors and Markov chain Monte Carlo settings
☒	☐	For hierarchical and complex designs, identification of the appropriate level for tests and full reporting of outcomes
☒	☐	Estimates of effect sizes (e.g. Cohen's *d*, Pearson's *r*), indicating how they were calculated

Our web collection on statistics for biologists contains articles on many of the points above.

Software and code

Policy information about availability of computer code

Data collection	L'image, no version # (L. Nittler, Carnegie Institution of Washington), Excel(Microsoft)
Data analysis	L'image; no version #(L. Nittler, Carnegie Institution of Washington), Qiime1;version 1.9.1(Caporaso et al., 2010), R; version 4.2.1 (R core Team, 2022), Excel; version 2209 (Microsoft), Ocean Data View; Version 2021(Schlitzer, 2021), ggplot2;version 3.3.6 (Wickham et al., 2009)

For manuscripts utilizing custom algorithms or software that are central to the research but not yet described in published literature, software must be made available to editors and reviewers. We strongly encourage code deposition in a community repository (e.g. GitHub). See the Nature Research guidelines for submitting code & software for further information.

Data

Policy information about availability of data

All manuscripts must include a data availability statement. This statement should provide the following information, where applicable:

- Accession codes, unique identifiers, or web links for publicly available datasets
- A list of figures that have associated raw data
- A description of any restrictions on data availability

All data is provided in manuscript and supplemental materials, with the exception of the raw nifH sequences, which were deposited in the Sequence Read Archive at National Center for Biotechnology Information (http://www.ncbi.nlm.nih.gov/sra) under Bioproject ID PRJNA730862. Additionally, data for Fig.1 and Fig.3 are available as a source data file with this paper.

Field-specific reporting

Please select the one below that is the best fit for your research. If you are not sure, read the appropriate sections before making your selection.

- [ ] Life sciences
- [ ] Behavioural & social sciences
- [x] Ecological, evolutionary & environmental sciences

For a reference copy of the document with all sections, see nature.com/documents/nr-reporting-summary-flat.pdf

Ecological, evolutionary & environmental sciences study design

All studies must disclose on these points even when the disclosure is negative.

Study description	N2 fixation rates were calculated from 7 locations across the North Pacific Subtropical Gyre at 15 m depth using dual-isotope incubations of 15N2 gas and 13C-bicarbonate. Each location had a natural-light treatment (natural light/dark cycle) and an all-dark treatment (24-hours of darkness) as a factorial design structure. Each treatment consisted of biological triplicates. A single subsample of the natural-light treatment from each location was used for cell-specific N2 fixation rate measurements by nanoSIMS. The one sample was analyzed with nanoSIMS at over 150 (~30 x 30 µm) analysis frames from each location. Replicate DNA samples from the seven locations were amplified for the nifH gene and sequenced. Sequences were pooled for average relative abundance.
Research sample	Samples were chosen from the across the North Pacific Subtropical Gyre as it has low nutrients and is know to have nitrogen fixation. The samples represent populations of plankton microorganisms between 0.2 and 210 micrometers. Samples were collected periodically across the Pacific to cover a broad geography and environmental conditions and increase the probability of encountering non-cyanobacterial N2 fixing organisms. Isotopically labeled (15N2, 13C-bicarbonate) planktonic community incubations from the 7 locations across the North Pacific Subtropical Gyre. Isotope incubations were filtered onto ~0.7 µm (GF/F, community N2 fixation) and 0.2 µm (silver, cell-specific N2 fixation) pore size filters for analysis. In addition, corresponding planktonic samples without isotope additions were collected and filtered (0.2 µm) for nifH gene amplification and sequencing.
Sampling strategy	A sample size of 4L for isotope incubations was chosen to ensure enough biomass (>10 µg N) was collected on the filter to accurately quantify 15N enrichment from particulate matter. Previous studies in similar nutrient-poor systems determined the minimum biomass necessary for reliable quantification (White et al., 2020). In addition, Triplicate incubations were chosen for a more robust average value and associated error. Subsamples for nanoSIMS analysis ranged from 0.1 to 0.5 L of incubation volume from each location. Based on the maximum abundances of the cells of interest (non-cyanobacterial diazotroph) in similar habitats (103 cells L-1), this volume would be sufficient while minimizing fixative waste used in sample preparation.
Data collection	While at sea, data was collected in recorded in the cruise-specific laboratory notebook by K.H., E.W.K.M., and K.A.T.K. Data were compiled into Excel spreadsheets by K.A.T.K. NanoSIMS data was automatically saved to internal servers by X.M., P.K.W., and K.H. at Lawrence Livermore National Laboratory. All analyzed nanoSIMS data was collected in excel spreadsheets by K.H. IRMS data was collected at the Isotope Biochemistry Laboratory in Hawaii and analyzed in excel by K.H. MIMS data was collected the University of California, Santa Cruz and analyzed in excel by K.H. Raw nifH sequence data was collected at the University of Illinois Core Facility and transferred via BaseSpace to K.A.T.K who analyzed and processed sequences in a remote server.
Timing and spatial scale	Samples were collected aboard the R/V Sally Ride in a relocation transect across the North Pacific from Guam to San Francisco. Samples were collected from November 5 to November 26, 2019. Seven samples were chosen across the Pacific in total covering approximately 5000 nautical miles, with 400-700 nautical miles between each sample. The large spatial scale was chosen to investigate as much area and different systems as possible provided the limitations set by nanoSIMS instrument time.
Data exclusions	Raw sequencing data was excluded during sequence analysis if it did not pass quality controls, which included removing: chimeric sequences, singletons, OTUs with less than 10 read counts, any non-diazotrophic sequences, and sequences with stop codons in the middle of the reading frame. No data was excluded from community N2 fixation rate measurements. NanoSIMS data were also removed if they did not pass quality controls. For nanoSIMS data, regions of interest with 15N enrichment values above the threshold (3x standard deviation of the standard cells) were discarded if the total rare isotopic counts were too low (< 100), the associated error was too high (> 30% to the 15N enrichment value), the ROI size was too small (< 0.4 µm diameter), lack of a clear cell-like outline in either 12C2- or 14N12C- or 15N enrichment was not measured in the majority of the defined ROI. Additionally, all ROIs, enriched and unenriched, were excluded if they did not meet minimum count threshold in 12C2- or 14N12C-.
Reproducibility	Reproducibility in a dynamic environment such as the surface ocean is nearly impossible, and our results represent a snapshot of an ever-changing system. Furthermore, Diazotroph distribution and the N2 fixation activity are dependent on factors specific to each location. However, isotope incubations and subsequent nanoSIMS analysis were conducted at 7 distinct locations, and N2-fixing cells were found at 6 out of 7 stations which validates the reproducibility of our results within that system.
Randomization	Randomization did not apply to this study. Samples were not grouped.
Blinding	Blinding was not necessary for this study. Cells were considered enriched and fixing N2 if the 15N enrichment value was higher than 3x the standard deviation of regularly used standards. This binary definition of enrichment does not allow for bias results.

Did the study involve field work? [x] Yes [ ] No

Field work, collection and transport

Field conditions	Study was ship based aboard the R/V Sally Ride. Sea Surface temperatures ranged from 15 to 30 C and salinity ranged from 33 to 35.5 PSU
Location	North Pacific Subtropical Gyre at sea level between 19.90 N, 153.77 E and 33.33 N, 144.98 W sampling a water depth of 15m.
Access & import/export	We used a ship to access open ocean habitats. Nitrogen fixation classically occurs in nutrient deplete ocean water which is found away from coastal setting. Sample were collected on the ship and stored on board until scientists arrived in San Francisco when the samples were transferred by personal truck to UC Santa Cruz. No permits were necessary for water sample collection while on board.
Disturbance	The R/V Sally Ride was on a relocation cruise from Guam to San Francisco, environment disturbance was minimal with less than 1 hour stop at each location.

Reporting for specific materials, systems and methods

We require information from authors about some types of materials, experimental systems and methods used in many studies. Here, indicate whether each material, system or method listed is relevant to your study. If you are not sure if a list item applies to your research, read the appropriate section before selecting a response.

Materials & experimental systems

n/a	Involved in the study
☒	☐ Antibodies
☒	☐ Eukaryotic cell lines
☒	☐ Palaeontology and archaeology
☒	☐ Animals and other organisms
☒	☐ Human research participants
☒	☐ Clinical data
☒	☐ Dual use research of concern

Methods

n/a	Involved in the study
☒	☐ ChIP-seq
☒	☐ Flow cytometry
☒	☐ MRI-based neuroimaging
